# Supplementary material for: Development and Validation of an Eating-Related Eco-Concern Questionnaire
Source: Nutrients. 2022 Oct 27;14(21):4517. doi: 10.3390/nu14214517 (PMC9658603; doi:10.3390/nu14214517)

Supplementary Material (S1). Eating-Related Eco-Concern Questionnaire.

Instruction: Please read each item and select the option that best describes how frequently each statement applied to you during the **PAST MONTH**, including today. There are no right or wrong answers.

The rating scale is as follows: 1 = Never; 2 = Rarely; 3 = Sometimes; 4 = Often; 5 = Always.

**Item**	**Never 1**	**Rarely 2**	**Sometimes 3**	**Often 4**	**Always 5**
1. I spend more time than other people searching for sustainable food.					
2. I avoid eating meat due to concerns about climate change.					
3. I avoid eating any animal products due to my concerns about climate change.					
4. I try not to waste food due to concerns about climate change.					
5. I actively encourage others to change their behaviors to slow climate change.					
6. I try to eat less because of my concerns about climate change.					
7. I avoid genetically modified foods due to concerns about biodiversity loss.					
8. I try to only eat organic foods or food produced without pesticides.					
9. I avoid foods that come with excess or non-recyclable packaging.					
10. I pay close attention to information on the impact that certain foods have on the environment (e.g., overfishing, greenhouse gasses, irrigation).					

Table S1. Descriptive statistics for climate change worry and eating-related eco-concern scores, and for the EDE-Q global and subscale scores in participants whose EDE-Q global scores were below clinical cut-off values (*n*=178).

**Measure**	**Mean (SD)**	**Range**
Climate change worry	29.44 (8.08)	10-49
Eating-related eco-concern	24.42 (7.63)	10-46
EDE-Q Global	1.30 (1.00)	0.00-3.89
Restraint	1.10 (1.21)	0.00-4.60
Eating concern	0.52 (0.84)	0.00-4.80
Weight concern	1.65 (1.30)	0.00-4.80
Shape concern	1.92 (1.41)	0.00-5.38

Note: EDE-Q = Eating Disorder Examination-Questionnaire; SD = standard deviation.

Table S2. Descriptive statistics and factor loadings for items included in the Eating-Related Eco-Concern Scale in participants whose EDE-Q global scores were below clinical cut-off values (*n*=178).

Item	Mean (SD)	Factor Loading
1. I spend more time than other people searching for sustainable food.	2.40 (1.02)	0.72
2. I avoid eating meat due to concerns about climate change.	2.75 (1.38)	0.72
3. I avoid eating any animal products due to my concerns about climate change.	1.94 (1.11)	0.67
4. I try not to waste food due to concerns about climate change.	3.16 (1.27)	0.61
5. I actively encourage others to change their behaviors to slow climate change.	2.63 (1.08)	0.63
6. I try to eat less because of my concerns about climate change.	1.53 (0.82)	0.54
7. I avoid genetically modified foods due to concerns about biodiversity loss.	1.79 (1.08)	0.61
8. I try to only eat organic foods or food produced without pesticides.	2.48 (1.18)	0.55
9. I avoid foods that come with excess or non-recyclable packaging.	2.91 (1.05)	0.69
10. I pay close attention to information on the impact that certain foods have on the environment (e.g., overfishing, greenhouse gasses, irrigation).	2.84 (1.06)	0.79

Note: SD = standard deviation. The score for each item ranges from *1 = Never to 5 = Always*.

Table S3. Associations among climate change worry, eating-related eco-concern, and disordered eating in participants whose EDE-Q global scores were below clinical cut-off values (n=178).

**Association between climate change worry and eating-related eco-concern**			
Predictor	*β (SE)*	*t (df)*	*p*
Climate change worry	0.52 (0.06)	8.63 (1)	**<.0001**
**Associations between each disordered eating characteristic and climate change worry**			
Predictor	*β (SE)*	*t (df)*	*p*
EDE-Q Global	0.31 (0.61)	0.51 (1)	.6079
Restraint	-0.36 (0.51)	-0.70 (1)	.4854
Eating concern	0.55 (0.73)	0.75 (1)	.4548
Weight concern	0.38 (0.47)	0.82 (1)	.4159
Shape concern	0.37 (0.43)	0.85 (1)	.3950
**Associations between each disordered eating characteristic and eating-related eco-concern**			
Predictor	*β (SE)*	*t (df)*	*p*
EDE-Q Global	0.69 (0.57)	1.21 (1)	.2268
Restraint	-0.28 (0.48)	-0.58 (1)	.5607
Eating concern	0.83 (0.69)	1.20 (1)	.2302
Weight concern	0.75 (0.44)	1.72 (1)	.0879
Shape concern	0.66 (0.40)	1.65 (1)	.1013

Note: df = degree of freedom; SE = standard error. Age was adjusted in all models. Significant results ($p < .05$) are bolded.

Figure S1. Histograms for (a) the Eating-Related Eco-Concern (EREC) score; (b) the Climate Change Worry Scale (CCWS) score; (c) Eating Disorder Examination-Questionnaire (EDE-Q) global score; (d) EDE-Q restraint subscale score; (e) EDE-Q eating concern subscale score; (f) EDE-Q weight concern subscale score; and (g) EDE-Q shape concern subscale score.

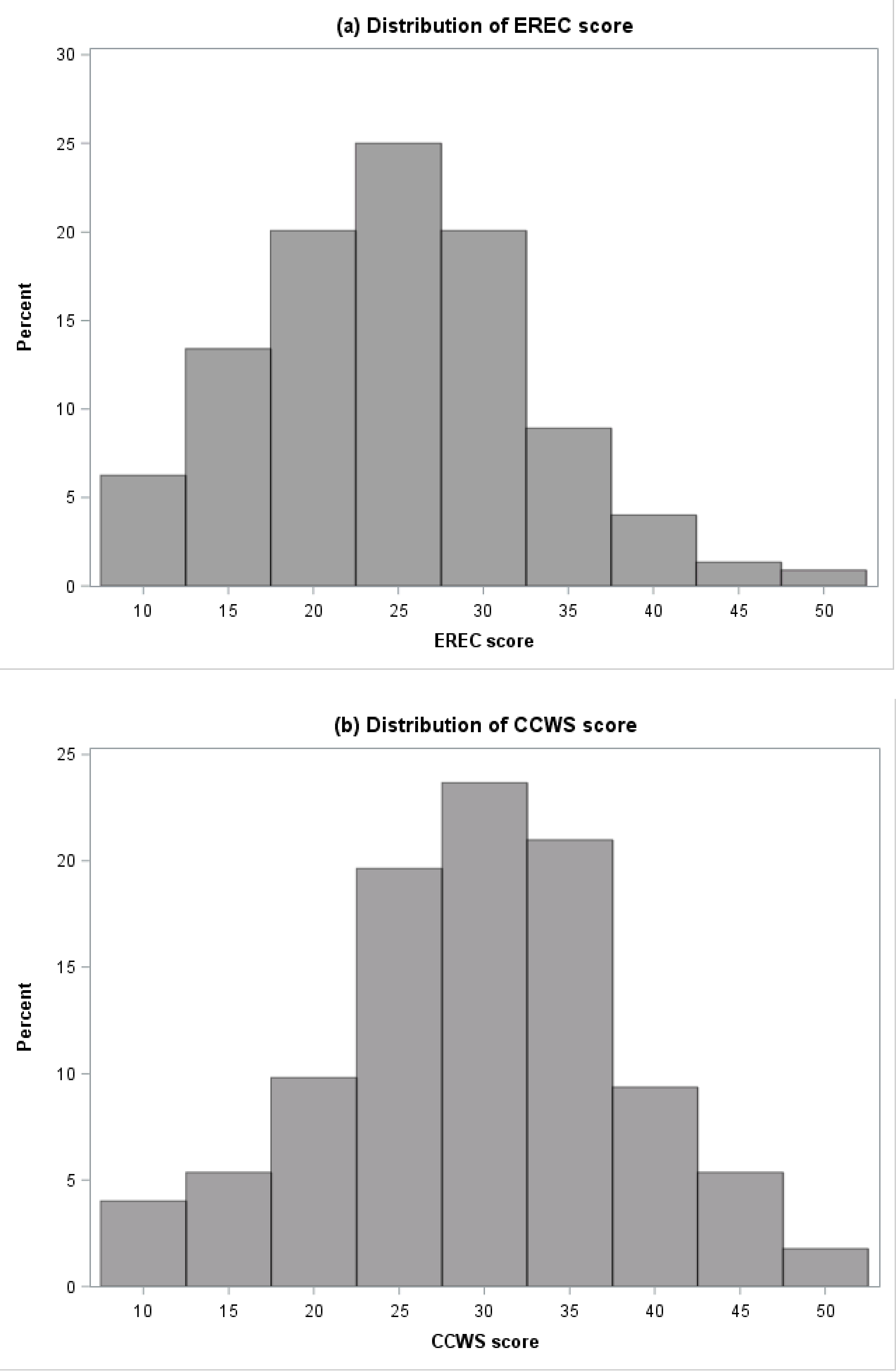

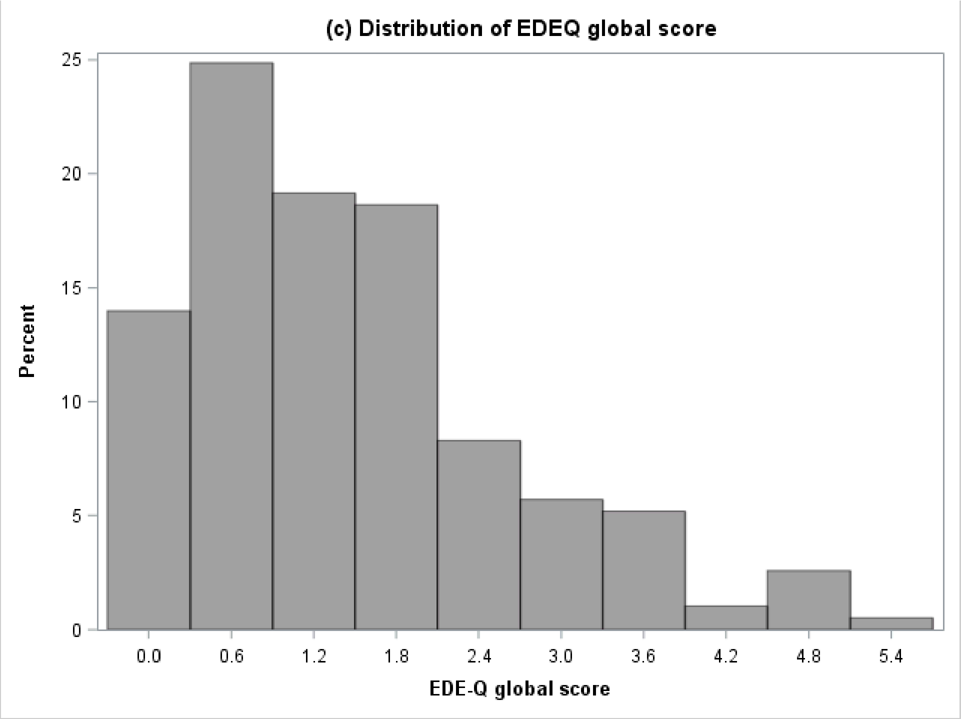

(c) Distribution of EDEQ global score
Percent
25
20
15
10
5
0
0.0
0.6
1.2
1.8
2.4
3.0
3.6
4.2
4.8
5.4
EDE-Q global score

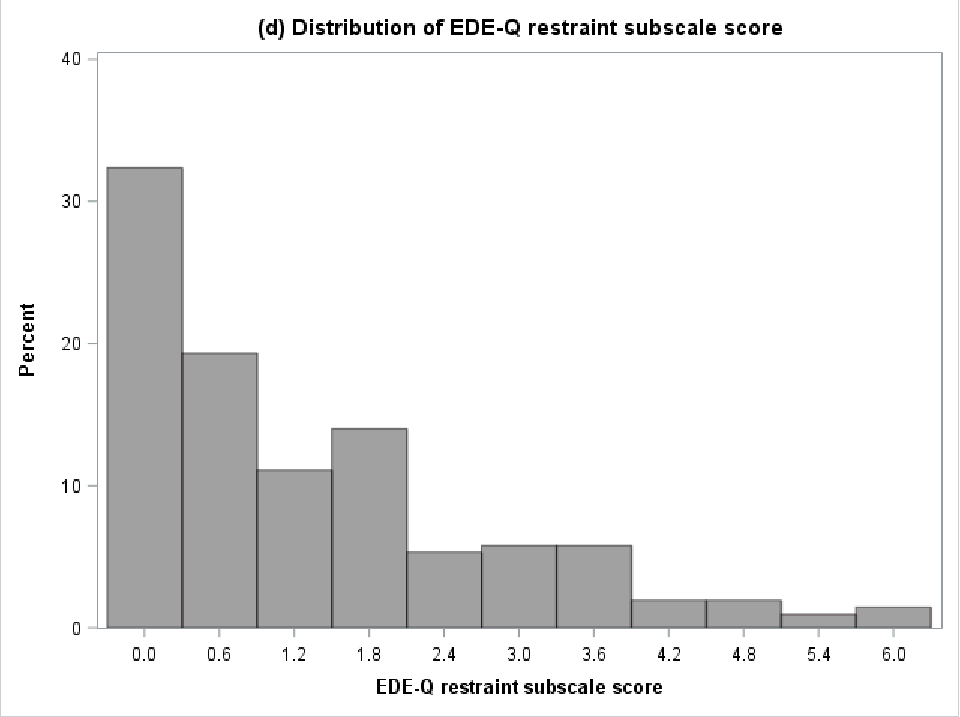

(d) Distribution of EDE-Q restraint subscale score
Percent
40
30
20
10
0
0.0
0.6
1.2
1.8
2.4
3.0
3.6
4.2
4.8
5.4
6.0
EDE-Q restraint subscale score

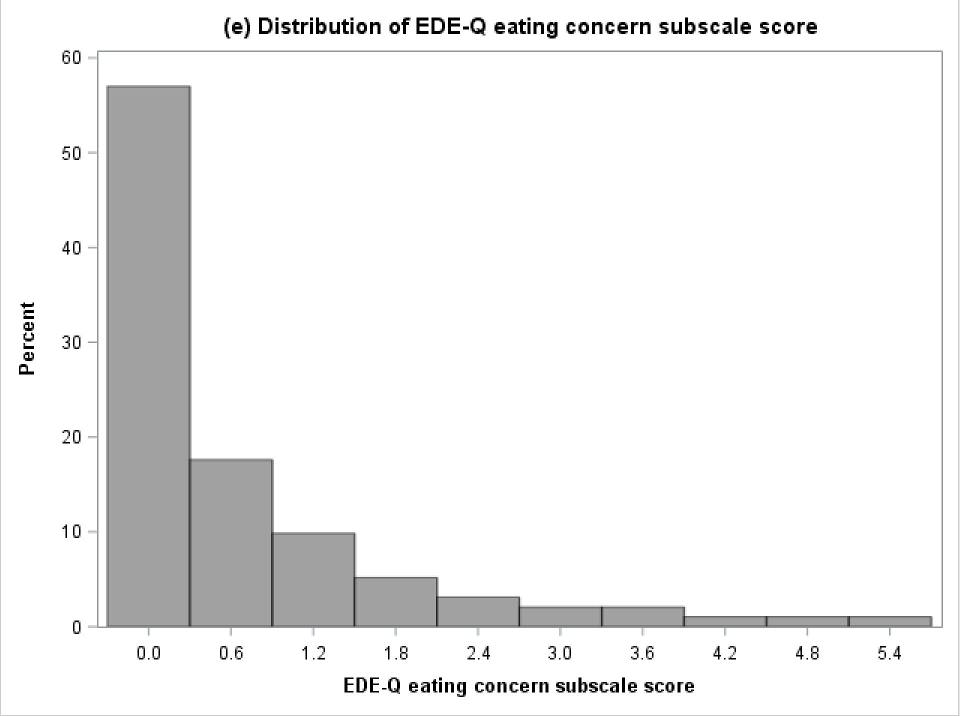
(e) Distribution of EDE-Q eating concern subscale score
Percent
60
50
40
30
20
10
0
0.0
0.6
1.2
1.8
2.4
3.0
3.6
4.2
4.8
5.4
EDE-Q eating concern subscale score

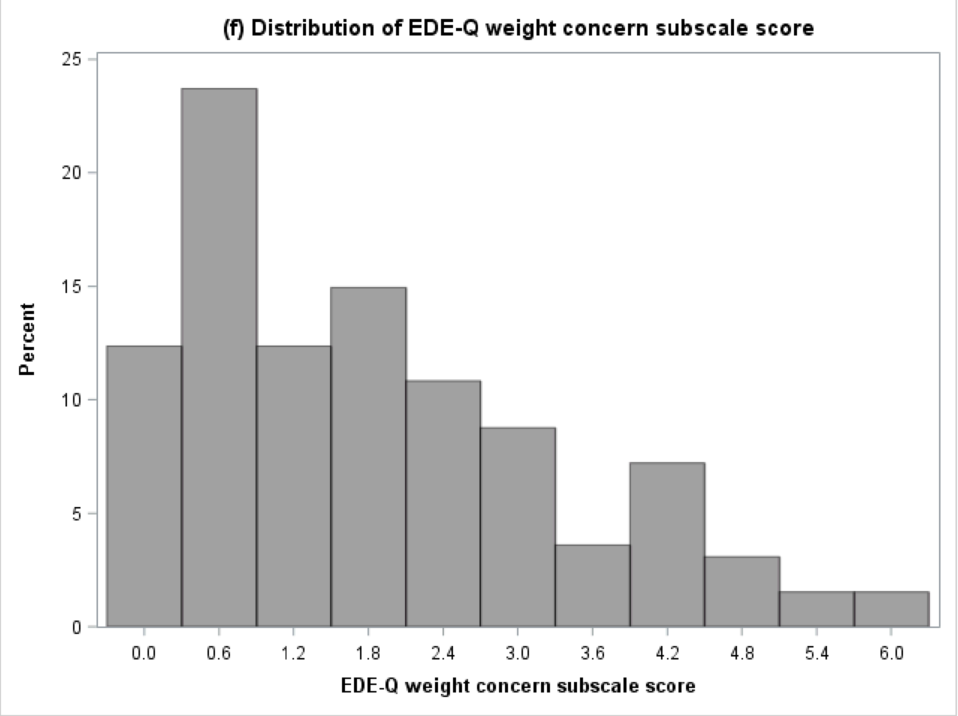
(f) Distribution of EDE-Q weight concern subscale score
Percent
25
20
15
10
5
0
0.0
0.6
1.2
1.8
2.4
3.0
3.6
4.2
4.8
5.4
6.0
EDE-Q weight concern subscale score

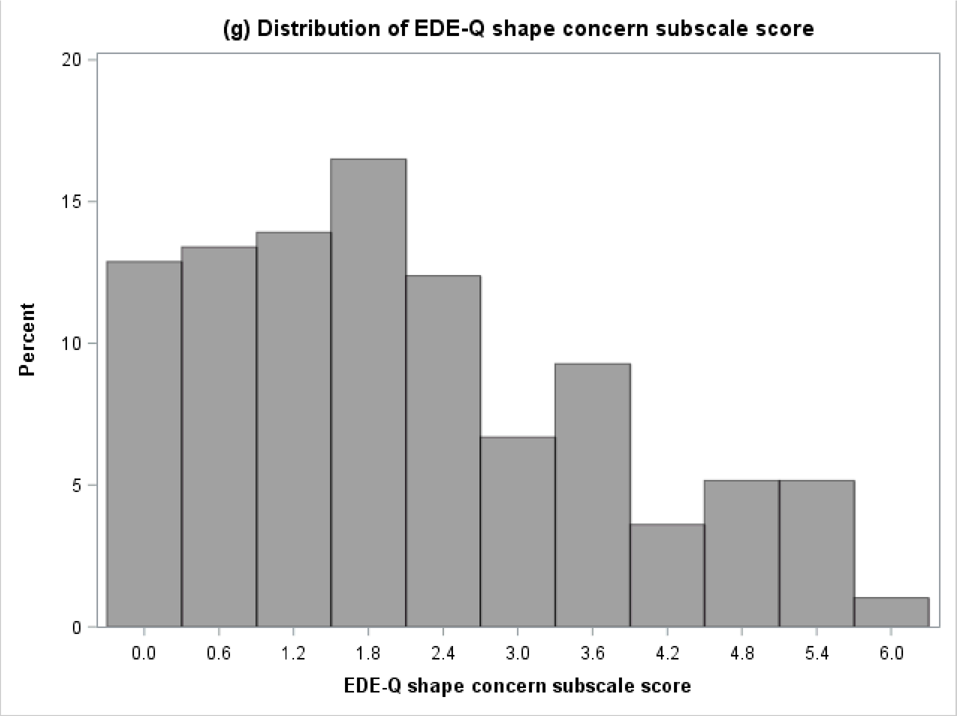
(g) Distribution of EDE-Q shape concern subscale score
Percent
20
15
10
5
0
0.0
0.6
1.2
1.8
2.4
3.0
3.6
4.2
4.8
5.4
6.0
EDE-Q shape concern subscale score

Figure S2. Bland-Atman plot for (a) the Eating-Related Eco-Concern (EREC) scale and the Climate Change Worry Scale (CCWS) and (b) the EREC scale (%) and the Eating Disorder Examination-Questionnaire (EDE-Q) global score (%) in participants whose EDE-Q global scores were below clinical cut-off values (n=178).

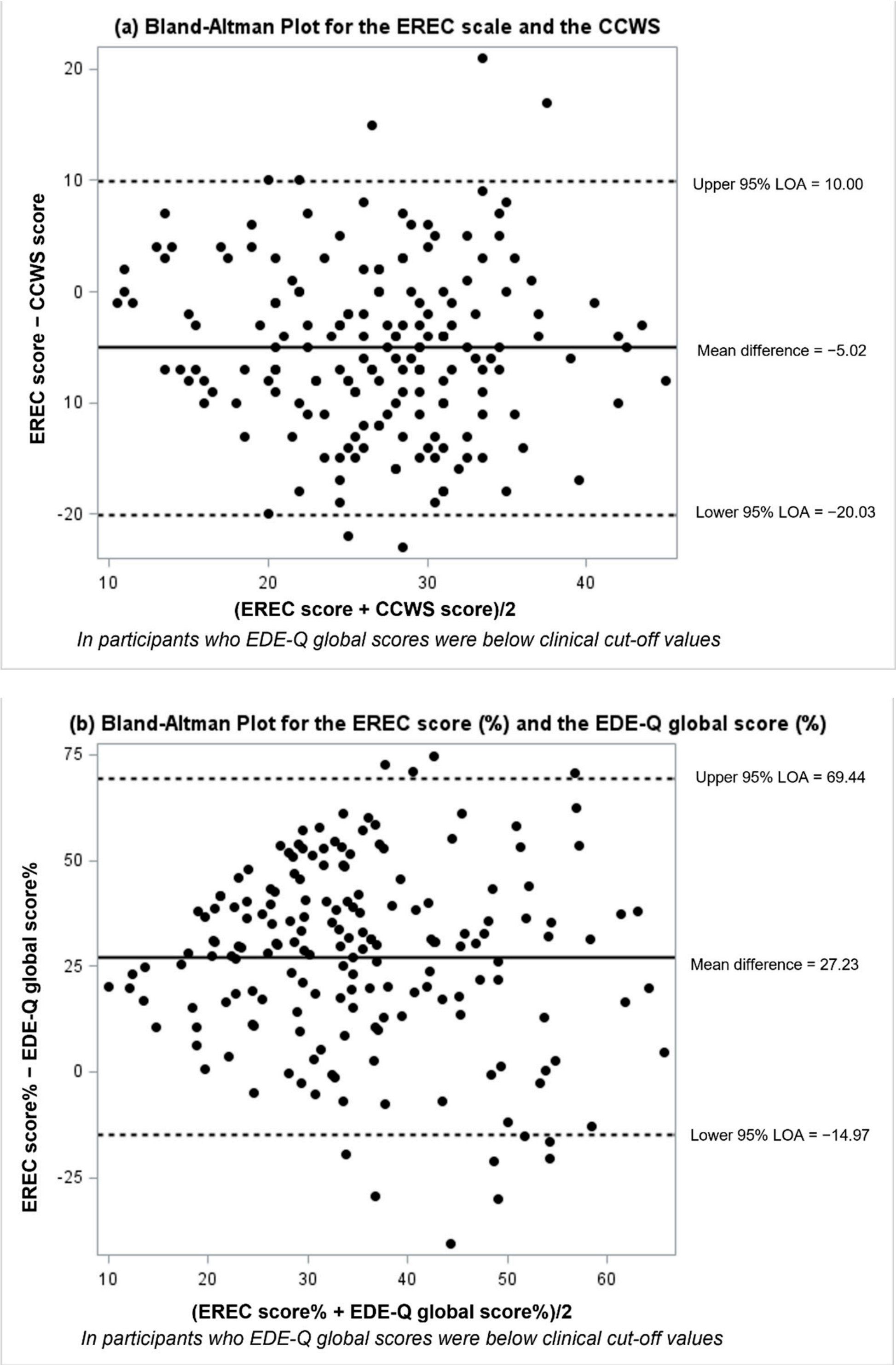

Supplement: Supplementary file 1 [file nutrients-14-04517-s001.zip › nutrients-1920534-supplementary.pdf]
